# Formulation of Genistein-HP β Cyclodextrin-Poloxamer 188 Ternary Inclusion Complex: Solubility to Cytotoxicity Assessment

**DOI:** 10.3390/pharmaceutics13121997

**Published:** 2021-11-24

**Authors:** Ameeduzzafar Zafar, Nabil K. Alruwaili, Syed Sarim Imam, Omar Awad Alsaidan, Faisal K. Alkholifi, Khalid Saad Alharbi, Ehab M. Mostafa, Abdullah S. Alanazi, Sadaf Jamal Gilani, Arafa Musa, Sultan Alshehri, Alenazy Rawaf, Ali Alquraini

**Affiliations:** 1 Department of Pharmaceutics, College of Pharmacy, Jouf University, Sakaka 72341, Al-Jouf, Saudi Arabia; nkalruwaili@ju.edu.sa (N.K.A.); osaidan@ju.edu.sa (O.A.A.); 2 Department of Pharmaceutics, College of Pharmacy, King Saud University, Riyadh 11451, Saudi Arabia; salshehri1@ksu.edu.sa; 3 Department of Pharmacology, College of Pharmacy, Prince Sattam Bin Abdulaziz University, Al Kharj 11942, Saudi Arabia; f.alkholifi@psau.edu.sa; 4 Department of Pharmacology, College of Pharmacy, Jouf University, Sakaka 72341, Al-Jouf, Saudi Arabia; kssalharbi@ju.edu.sa; 5 Department of Pharmacognosy, College of Pharmacy, Jouf University, Sakaka 72341, Al-Jouf, Saudi Arabia; emmoustafa@ju.edu.sa (E.M.M.); akmusa@ju.edu.sa (A.M.); 6 Department of Clinical Pharmacy, College of Pharmacy, Jouf University, Sakaka 72341, Al-Jouf, Saudi Arabia; Asdalananzi@ju.edu.sa; 7 Health Sciences Research Unit, Jouf University, Sakaka 72341, Al-Jouf, Saudi Arabia; 8 Department of Basic Health Sciences, Preparatory Year, Princess Nourah Bint Abdulrahman University, Riyadh 11671, Saudi Arabia; SJGlani@pnu.edu.sa; 9 Department of Medical Laboratory, College of Applied Medical Sciences-Shaqra, Shaqra University, Shaqra 11961, Saudi Arabia; Ralenazy@su.edu.sa; 10 Department of Pharmaceutical Chemistry, Faculty of Clinical Pharmacy, Al Baha University, Al Baha 65511, Saudi Arabia; aalquraini@bu.edu.sa

**Keywords:** genistein, Hydroxypropyl β cyclodextrin, poloxamer, cytotoxicity, antioxidant, antimicrobial activity

## Abstract

The current study was designed to prepare the inclusion complex Genistein (GS) using Hydroxypropyl β cyclodextrin (HP β CD) and poloxamer 188 (PL 188). The binary inclusion complex (GS BC) and ternary inclusion complex (GS TC) were developed by microwave irradiation technique and evaluated for a comparative dissolution study. Further, the samples were assessed for FTIR, DSC, XRD, and NMR for the confirmation of complex formation. Finally, antioxidant and antimicrobial studies and cytotoxicity studies on a breast cancer (MCF-7) cell line were conducted. The dissolution study result showed a marked increment in GS dissolution/release after incorporation in binary (GS: HP β CD, 1:1) and ternary (GS: HP β CD: PL 188; 1:1:0.5) inclusion complexes. Moreover, the ternary complex exhibited a significant enhancement (*p* < 0.05) in dissolution than did the binary complexes. This might be due to the presence of PL 188, which helps in solubility enhancement of GS. DSC, XRD and SEM evaluation confirmed the modification in the structure of GS. FTIR and NMR results indicated the formation of an inclusion complex. The antioxidant and antimicrobial activity results revealed that GS TC has shown significant (*p* < 0.05) higher activity than pure GS. The cytotoxicity study results also depicted concentration-dependent cytotoxicity. GS TC exhibited significantly (*p* < 0.05) high cytotoxicity to cancer cells (IC_50_ = 225 µg/mL) than pure GS (IC_50_ = 480 µg/mL). Finally, it was concluded that a remarkable enhancement in the dissolution was observed after the inclusion of GS in the ternary complex and it therefore has significant potential for the treatment of breast cancer.

## 1. Introduction

Worldwide, breast cancer is one of the most common cancers among women and the leading cause of death after lung cancer. The prognosis of this cancer is about five years in 75% of women [1]. Chemotherapy and radiation therapies are commonly used treatment modules for this type of cancer. Furthermore, the application of traditional chemotherapy and radiotherapy is somewhat limited owing to their cost, their side effects, as well as extra toxicity to untargeted tissues/organs [2].

About 40–70% of drugs exhibit poor solubility and belong to the BCS class II and IV category. Due to the low solubility and dissolution rate, the bioavailability is limited. Hence, the dissolution of active pharmaceutical ingredients is the major hurdle for designing a suitable drug delivery system [3]. Several techniques and polymer systems have been developed and reported in the literature to enhance drug solubility and bioavailability. These techniques are solid dispersion [4], inclusion complex [5,6], and micronization/nanonization [7].

**Figure 1:**
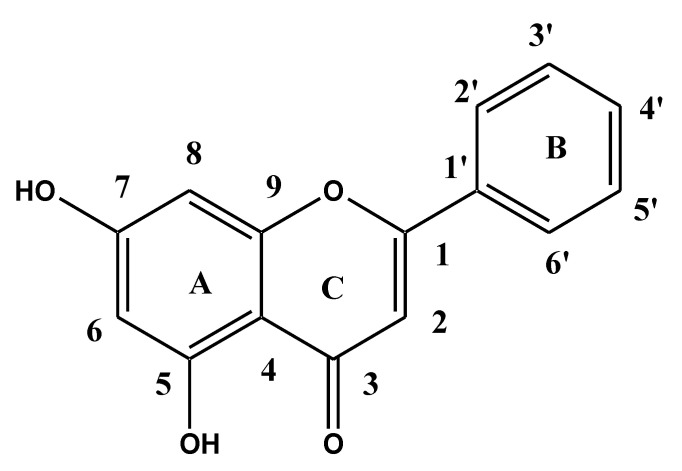
Chemical structure of genistein.

Genistein (GS, Figure 1) is an isoflavone aglycone obtained from soybean. It is used as a nutraceutical ingredient in food as well as in the pharmaceutical industry. Furthermore, it exhibited reduced bioavailability due to its limited solubility (1.43 µg/mL) [8], first-pass metabolism by glucuronidation and sulfation [9]. Many strategies have been applied to overcome the problem associated with GS, including nanoparticles [10,11,12], cyclodextrin complex [13], amylose complexes [14], Zein NPs [15], Zein/Carboxymethyl cellulose-chitosan coated NPs [10], micelles [8], NLCs [9], and eudragit-NPs [16].

Hydroxypropyl β cyclodextrin (HP-β-CD) is a cyclic oligosaccharide compound and it is obtained from the β-cyclodextrin by incorporation of hydroxyl propyl group on reaction with propylene oxide [17]. The benefit of adding the hydroxyl propyl to cyclodextrin (β-CD) is to enhance the solubilizing property (around 27 folds higher than β CD). HP-β-CD has a hydrophobic center with an outer hydrophilic surface (6.0–6.5 Å) [18]. It is widely used to improve the solubility of poorly water-soluble drugs. The US Food and Drug Administration (FDA) has recommended the use of HP-β-CD as a solubilizer [19].

Various research work has been reported on inclusion complexes using the cyclodextrin and ternary substance [6,7,19]. Shah et al., formulate inclusion complex of cefuroxime axetil using HP β cyclodextrin and different ternary substances i.e., poloxamer, HPMC, PEG 400 and PVP K30. The ternary inclusion complex exhibited a high stability constant as compared to the binary inclusion complex [20]. Alshehri et al., prepared ternary inclusion complex of piperine using cyclodextrin and Hydroxypropyl methyl cellulose [21]. The ternary inclusion complex depicted significant enhancement in the solubility and dissolution of piperine as compared to pure piperine and binary inclusion complex. Soe et al. developed the binary and ternary inclusion complex of asiaticoside using cyclodextrin and polymers (poloxamer and chitosan). The ternary complex has shown higher solubility, dissolution and permeation data than the binary complex [22].

Poloxamer is a nontoxic linear, non-ionic co-polymer approved by the FDA for application in pharmaceutical products [23]. It contains two hydrophilic parts which are linked with the lipophilic part. It has good surfactant properties and is commonly used for solubility enhancement of poorly soluble drugs [23,24].

To date, no formulation of GS with HP-β-CD and PL 188 has been reported in the literature for the enhancement of solubility and in vitro activity of GS. The present research work was designed to prepare a GS inclusion complex using HP-β-CD and PL 188. The inclusion complex was developed by microwave technique and evaluated for the study of its solubility and dissolution. The inclusion complex was further characterized by FTIR, DSC, XRD, and NMR. Finally, a cytotoxicity study (MCF-7 cell line), antioxidant study, and antimicrobial study was conducted to evaluate the effect of the inclusion complex.

## 2. Materials and Methods

### 2.1. Materials

Genistein (GS), Poloxamer 188 (PL 188), methanol, ethanol and acetonitrile were procured from Sigma Aldrich (St. Louis, MA, USA). Hydroxyl propyl beta-cyclodextrin (HP-β-CD) was procured from SD Fine Chemical (Mumbai, India). The other chemicals used in the study that were obtained from the pharmaceutics laboratory are analytical grade.

### 2.2. Methods

#### 2.2.1. HPLC Method of Genistein

The analysis of GS was done previously with the developed HPLC method with slight modification [25]. An HPLC system (Shimadzu, Kyoto, Japan) with a SPA 20 A UV detector, C_18_ column (5 µm particle size, 250 mm × 4.6 mm internal diameter) was used. The study was performed at 250 nm with flow rate of 0.75 mL/min. The mobile phase contains acetonitrile: water with 0.05% trifluoroacetic acid (7:3) was used for analysis. The study was conducted at 25 °C using the sample injection volume of 20 µL.

#### 2.2.2. Phase Solubility Study

A phase solubility study was performed by the supersaturation method. The excess amount of GS was added into different concentrations of an aqueous solution of HP-β CD for binary and HP-β CD with PL 188 for the ternary system. The sample was kept in a water bath shaker (Thermo Fisher Scientific, Mumbai, India) for 72 h at 37 ± 0.5 °C. The sample was centrifuged at 5000 rpm for 15 min and the supernatant was collected, filtered through a micron filter (0.25 µm), and diluted. The concentration was analyzed by the previously developed HPLC method with a slight variation [25]. The sample (20 µL) was injected into column for determination of concentration. The solubility constant (Ks) and complexation efficiency (CE) was calculated by the given mathematical formula [26].

(1) $$\mathrm{Solubility}\;\mathrm{constant}\;\left(\mathrm{Ks}\right)=\frac{Slope}{So\;\left(1-Slope\right)}$$

(2) $$\mathrm{Complexation}\;\mathrm{efficiency}\;\left(\mathrm{CE}\right)=\frac{Slope}{\left(1-Slope\right)}$$

#### 2.2.3. Physical Mixture

The binary physical mixture (GS: HP-β CD as GS-BM) and ternary physical mixture (GS: HP-β CD: PL 188 as GS-TM) were prepared and their composition shown in Table 1. The weighed quantity of ingredients for GS-BM and GS-TM were taken and transferred into a mortar. The samples were properly mixed to get a uniform mixture. The physical mixture was passed through sieve number 80 to get uniform sized particles, and stored for further study.

#### 2.2.4. Binary and Ternary Inclusion Complex

The microwave technique was used to prepare GS-BC and GS-TC (Table 1). For the binary inclusion complex, the appropriate quantity of GS and HP-β-CD (1:1, molar ratio) was taken in a beaker and ethanol-water mixture was added to form a smooth paste. The smooth paste was kept in a microwave oven with a time interval of 1 min for 5 min at 600 W [21,27]. Similarly, the ternary inclusion complex was prepared by taking GS, HP-β CD, and PL 188 in the same condition for 4 min. The prepared inclusion complex was collected from the microwave and cooled down for pulverization. The sample was crushed to powder and passed through sieve number 80 for uniform and fine powder. The fine powder of inclusion complex is stored in a desiccator for further study [28].

#### 2.2.5. Saturation Solubility Study

The saturation solubility study of pure GS, physical mixture (GS-BM and GS-TM), and inclusion complex (GS BC and GS TC) were determined by the supersaturation method. The excess amount of each sample was added to a fixed volume of water and kept in a water bath shaker for 72 h at 37 ± 0.5 °C. The sample was centrifuged at 5000 rpm for 15 min and the supernatant was collected, filtered, and diluted to estimate the concentration of GS by developed HPLC method [25].

#### 2.2.6. Dissolution Study

The release study of the pure GS, physical mixture (GS-BM and GS-TM), and inclusion complex (GS-BC and GS-TC) was done in phosphate buffer (pH 6.8) using USP dissolution apparatus II (USP-II Sotex AG, Aesch, Switzerland). The release media (900 mL) was used to perform the study. The sample (5 mg of GS) was packed into a muslin cloth and tied with the paddle. The paddle was dipped in the dissolution medium with a rotation speed of 50 rpm. The released content (5 mL) was collected and the same volume of fresh buffer was replaced. The aliquot was filtered through a membrane filter and the GS concentration analyzed by the HPLC method [26]. The release data of GS TC was fitted to different release kinetic models to select the best fit model [8].

#### 2.2.7. Drug Content

The inclusion complex was weighed (5 mg of GS) and transferred into a glass vial. Methanol (5 mL) was added to dissolve the sample and then sonicated for 2 min. The solution was filtered and GS concentration was analyzed by previously developed HPLC method with slight variation [26].

#### 2.2.8. Fourier Transform Infra-Red (FTIR)

The FTIR spectra of Genistein (GS), HP-β Cyclodextrin (HP-β-CD), poloxamer 188 (PL 188), genistein physical mixture (GS-PM, GS: HP β CD: PL188), genistein binary complex (GS-BC), genistein ternary complex (GS-TC). were analyzed by the FTIR instrument (ATR-FTIR, Bruker Alpha, Ettlingen, Germany). The samples were scanned between 4000–500 cm^−1^. The spectra of pure GS were interpreted and compared with inclusion complex (GS BC and GS TC) to check the change in peak height and position.

#### 2.2.9. Differential Scanning Calorimetry (DSC)

The thermal analysis of GS, HP-β-CD, PL188, GS-PM, GS-BC, and GS-TC were done by using DSC (Mettler, Toledo, OH, USA). Each sample (5 mg) was packed individually into an aluminum pan and placed into the instrument. A reference standard pan was placed in instrument. The samples were scanned between 25–400 °C (heating rate 5 °C/min) under continuous nitrogen supply to maintain the inert condition.

#### 2.2.10. X-ray Diffraction Analysis

The XRD study of pure GS, HP-β-CD, PL188, GS-PM, GS-BC, and GS-TC was analyzed by an X-ray diffraction instrument (Ultima IV diffractometer, Rigaku Inc., Tokyo, Japan). The samples were placed into a sample holder and the spectra were recorded between 5 to 60° at 2 theta level with a scanning rate of 0.5°/min.

#### 2.2.11. Nuclear Magnetic Resonance (NMR)

NMR study was performed to evaluate the change in chemical shift of pure GS after preparation of the inclusion complex (GS-BC and GS-TC). ^13^C NMR spectral analysis of GS, GS-BC and GS-TC was performed using an NMR instrument (Bruker NMR; Bruker, Switzerland). The samples were dissolved in DMSO and their spectra were taken by using tetramethylsilane (TMS) as an internal standard. The study was performed at 176 MHz and the spectra collected using topspin 3.2 software.

#### 2.2.12. Scanning Electron Microscope (SEM)

The change in surface morphology of pure GS and inclusion complex (GS-TC) was evaluated using SEM (JSM 6360A, JOEL, Tokyo, Japan). The thin layer of GS and GS-TC were placed on a brass stub and fixed with double sided tape. The sample was coated with gold by using a sputter coater. The instrument was operated at 20 kV voltage and the images were captured with a high-resolution microscope.

#### 2.2.13. Antioxidant Activity

The in-vitro antioxidant activity of pure GS and ternary inclusion complex (GS-TC) was analyzed by the reported DPPH method with slight modification [29]. The standard stock solution of pure GS and GS TC (1 mg/mL) was prepared in methanol. Further, different concentrations between 10–100 µg/mL were prepared after dilution in methanol. 500 µL of each sample was added to 125 µL of freshly prepared DPPH solution (0.02% in methanol). The mixture was mixed and kept in a dark condition for 1 h. After complete reaction, the colour changes from violet to colourless indicating the antioxidant activity and analyzed by UV-spectrophotometer at 517 nm [30]. A control sample was also analyzed for the calculation of radical scavenging activity by a given mathematical equation:

(3) $$\%\;\mathrm{Redical}\;\mathrm{scavenging}\;=\frac{Abs\;of\;control\;sample-Abs\;of\;test\;sample}{Abs\;of\;control\;sample}\times 100$$

The antioxidant activity of the GS and GS-TC complex were further evaluated by the ABTS scavenging method using a slightly modified reported method [31]. The different concentration (5–100 µg/mL) of each sample (0.1 mL) was taken and mixed with ABTS solution (0.9 mL). The mixture was incubated for 30 min at room temperature. BHT was used as a standard for the determination of antioxidant potential. After completion of the reaction, the sample was measured at 734 nm. 

#### 2.2.14. Cytotoxicity Study

The breast cancer cell line MCF-7 was used for the cytotoxicity study of pure GS and GS-TC. The cell line was grown into a mammalian culture medium with a supply of supplements (10% fetal bovine serum, penicillin 100 U/mL, streptomycin 10 U/mL) and 5% CO_2_ at 37 ± 0.5 °C in a CO_2_ incubator (Galaxy^®^ 170R CO_2_ incubator, Eppendorf, Germany). GS and GS-TC were dissolved in DMSO and further diluted between 10–2000 µM with phosphate buffer. The pure GS and GS TC were added into a seeded well of the microplate (96-well plate) and further incubated for 72 h. The cell viability was determined by adding 3-(4,5-dimethylthiazol-2-yl)-2, 5-diphenyltetrazolium bromide (MTT) into each microplate of cells. Then, the formazan crystal was fully dissolved by adding 100 µL of DMSO and absorbance was analyzed at 490 nm using a microplate reader. The % inhibition was calculated by following the formula against control.

(4) $$\mathrm{Growth}\;\mathrm{inhibition}=\frac{\mathrm{OD}\;\mathrm{control}\;\mathrm{sample}-\mathrm{OD}\;\mathrm{test}\;\mathrm{sample}}{\mathrm{OD}\;\mathrm{control}\;\mathrm{sample}}\times 100$$

#### 2.2.15. Antimicrobial Activity

The antimicrobial study was done by a cup-pate method using the microorganisms *S. aureus* and *B. subtilis*. Muller-Hinton agar media was prepared and sterilized at 121 °C using an autoclave. *S. aureus* and *B. subtilis* were transferred in sterilized Petri plates with sterilized media under aseptic conditions. Petri plates were kept for 30 min to solidify the media and the wells were made with a sterilized borer. The pure GS, HP-β-CD-PL 188 and GS TC were solubilized in sterilized water and added to each well. The petriplates were incubated into an incubator (Thermo Fisher Scientific, Waltham, MA, USA) for 24 h at 37 ± 0.5 °C. The normal saline was used as a control and the zone of inhibition was measured by a graduated scale.

#### 2.2.16. Statistical Analysis

Graph Prism Pad InStat 3 software (San Diego, CA, USA) was used to evaluate the statistical analysis. The experimental results were compared at *p* < 0.05 to check the significant difference. One-way ANOVA and Tukey Kramer test was used to compare the results. All the data were performed in triplicate and presented as mean ± SD.

## 3. Result and Discussion

### 3.1. Phase Solubility Study

**Figure 2:**
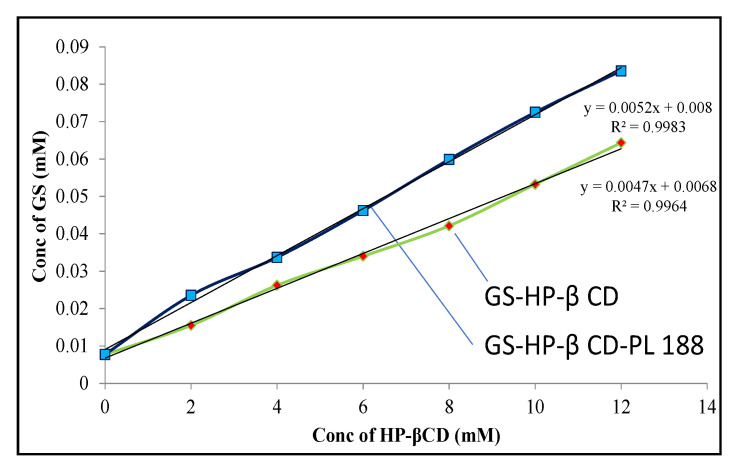
Phase solubility of Genistein—HP β CD and Genistein—HP β CD-PL 188.

The phase solubility study of GS in different concentrations of HP-β-CD (binary) and HP-β-CD—PL 188 (ternary) was determined to assess the affinity between drug and carrier as depicted in Figure 2. The solubility of GS increases with an increase in the HP β CD concentration and can classify as AL type of relation between a host-guest molecule [32]. The slope for the binary and ternary mixture was found to be 0.007 and 0.006 (<1), signifying the formation of a first-order complex. It also exhibited a 1:1 stoichiometric ratio between 0–12 mM concentration. The solubility of GS increases 8.32-fold in binary (17.39 µg/mL) and 10.32-fold in ternary (22.59 µg/mL) at 12 mM HP-β-CD than pure GS (2.09 µg/mL). The stability constant (Ks) was found to be 592.05 M^−1^ for binary and 1042.75 M^−1^ for the ternary complex. The high Ks value for the ternary complex represented more interaction of GS with the cavity of HP β CD. The ternary substance (PL 188) formed the network with the outer surface of HP-β-CD as well as with GS- HP-β CD and supported the formation of co-complexes [33,34]. The complexation efficiency (CE) for binary and ternary complexes was found to be 0.46 and 0.81, respectively. The higher value of CE for the ternary complex is due to the presence of PL 188, which helps to enhance the complex formation. Danciu et al. formulated GS inclusion complex using HP-β CD in a ratio of 1:1 and the result showed the stability constant value of 10.9 M^−1^ which is less than our developed GS-TC [35]. In another study, Anwer et al. formulated ternary inclusion complex of Arbidol hydrochloride using poloxamer 188 and showed better stability constant value of 2134 M^−1^ [24]. The findings of our study were found to be greater than the binary system and similar to the ternary systems.

### 3.2. Saturation Solubility Study

**Figure 3:**
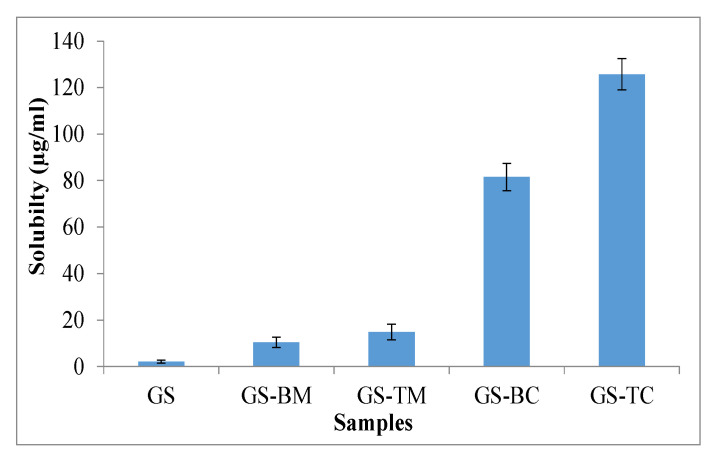
Saturation solubility of Genistein (GS), binary physical mixture (GS-BM), ternary physical mixture (GS-TM), genistein binary complex (GS-BC) and genistein ternary complex (GS-TC).

The saturated solubility of GS was found to be 2.09 µg/mL which is agreed with the reported value (2.03 µg/mL) [8]. The order of solubility found as follows GS TC > GS-BC > GS-TM > GS-BM > GS, as depicted in Figure 3. GS showed significant (*p* < 0.05) enhanced solubility in the physical mixture and inclusion complex. GS-BM and GS-TM showed 4.95 (10.34 ± 2.25 µg/mL) and 7.11 (14.86 ± 3.43 µg/mL) fold solubility enhancement than pure GS due to the presence of PL 188 (surfactant) as a ternary substance. However, in case of GS-BC and GS-TC, it exhibited 39.01 (81.54 ± 5.87 µg/mL) and 60.17-fold (125.76 ± 6.75 µg/mL) increments in aqueous solubility than pure GS. The significant (*p* < 0.001) high solubility of GS in ternary complex (GS-TC) is due to the addition of PL 188. It helps to enhance the solubility of GS by reducing the interfacial tension between GS and water [22,36]. Inoue et al. formulated binary inclusion complex using GS with γCD at a ratio of 1:1 and exhibited 51-fold higher solubility than pure GS, which is lower than GS-TC [37]. Our study results showed greater solubility enhancement than prepared binary system and reported GS with γCD complex.

### 3.3. Dissolution Study

**Figure 4:**
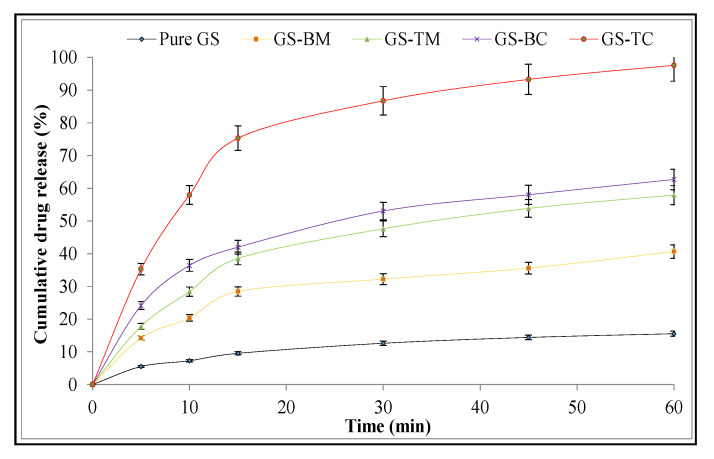
Dissolution study of Genistein (GS), binary physical mixture (GS-BM), ternary physical mixture (GS-TM), genistein binary complex (GS-BC) and genistein ternary complex (GS-TC). Difference was considered significant if *p* < 0.05.

Dissolution studies of pure GS, physical mixture (GS-BM and GS-TM), and inclusion complexes (GS-BC and GS-TC) were performed using a dissolution apparatus and the data was represented graphically (Figure 4). The order of GS release followed as GS-TC > GS-BC > GS-TM > GS-BM > GS. GS exhibited 15.55 ± 2.23% release in 1 h, whereas GS-BM and GS-BC showed 40.64 ± 4.76% and 57.9 ± 4.8% release, respectively. The high release of GS is due to the complex formation with HP-β CD leads to a reduction of GS crystallinity due to conversion into an amorphous form. GS-TC exhibited significant (*p* < 0.001) higher release (97.61 ± 4.88% in 1 h) than GS-TM (62.68 ± 5.01% in 1 h). The high release from the ternary complex is due to the presence of a ternary substance (PL 188). HP-β CD completely diminishes the crystallinity of GS and converts to an amorphous form. The presence of PL 188 reduces the interfacial tension between drug and dissolution media [38]. The ternary substance (PL 188) gives the synergistic effect with HP-β CD and enhances the dissolution rate of GS in GS-TC [39]. These findings indicate that the complexation with HP-β CD of low soluble therapeutics can be an effective formulation approach to augment solubility and release rates. Anwer et al. formulated a ternary inclusion complex of Arbidol hydrochloride using β CD with ternary substance poloxamer 188. The dissolution result revealed 94.5% drug release in the tested 1 h [24]. Our dissolution results also showed a similar type of result using PL 188 as ternary substance. The release data of GS-TC was fitted into a zero-order, first-order, Higuchi and Korsmeyer-Peppas kinetic release model. The Korsmeyer-Peppas model was found to be the best fit model due to its maximum R2 (0.9235) [40]. The n value was found to be 0.36 (less than 0.45) and indicated the release mechanism from GS-TC was a fickian type of diffusion.

### 3.4. Drug Content

The drug content of GS-BC and GS-TC was determined and found to be 98.95 ± 3.98% and 99.72 ± 3.11%, respectively. It indicates that GS was completely incorporated and uniformly distributed into the prepared inclusion complex.

### 3.5. Fourier Transform Infra-Red

**Figure 5:**
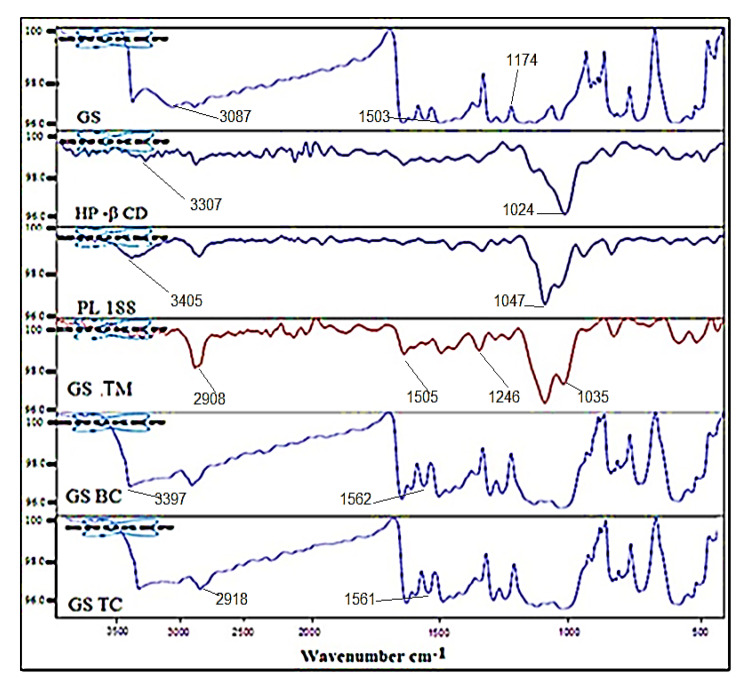
IR spectra of Genistein (GS), HP-β Cyclodextrin (HP-β CD), poloxamer 188 (PL 188), genistein ternary mixture (GS-TM), genistein binary complex (GS-BC), genistein ternary complex (GS-TC).

The FTIR spectroscopy of GS, HP-β CD, PL188, GS-PM, GS-BC, and GS-TC were analyzed by FTIR instrument and spectra depicted in Figure 5. GS showed the characteristic peak at 3087 cm^−1^ (phenolic OH) and 1503 cm^−1^ of carbonyl stretching vibration. The other characteristic peak was found for stretching vibration at 1174 cm^−1^ of aromatic C=C assuring the authenticity of GS [41]. The spectra of HP-β CD showed the characteristic peaks at 1024 and 3307 cm^−1^ for C-O-C and hydroxyl groups, respectively. PL188 exhibited characteristic peaks at 1047 and 3405 cm^−1^ for the same functional group. However, the spectra of GS-TM exhibited slight variations from pure GS and carrier peaks. It was worthy to note that all the three characteristics hydroxyl group peaks of pure GS, HP-β CD and PL 188 were observed at 2908, 3314 and 3400 cm^−1^, respectively. The spectra of GS-TM also comply with slight changes for the functional group C=O and aromatic C=C of pure GS at 1505 and 1246 cm^−1^. Correspondingly, the peaks in GS-TM were also observed with minute change for C-O-C of the carrier HP-β CD at 1035 and PL 188 at 1106 cm^−1^. In GS BC spectra, the characteristic stretching vibration peaks of the pure GS were found at 3397 and 1562 cm^−1^ for the hydroxyl and carbonyl group. However, GS-TC spectra have also shown the stretching vibration peak with slight changes at 2918 and 1561 cm^−1^ for the hydroxyl and carbonyl group. The change in spectral peak confirms the formation of inclusion complex.

### 3.6. Differential Scanning Calorimetry

**Figure 6:**
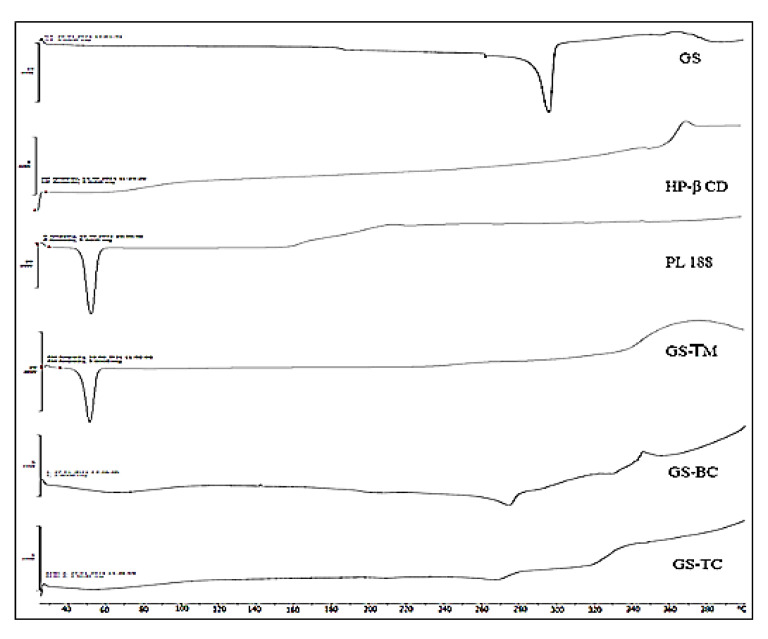
DSC thermogram of Genistein, HP β CD, poloxamer 188, genistein ternary mixture (GS TM), genistein binary complex (GS BC) and genistein ternary complex (GS TC).

Figure 6 shows the DSC spectra of pure GS, HP-β CD, PL188, GS-PM, GS-BC, and GS-TC. The pure GS spectra showed the sharp characteristic peak at its melting point 298 °C. The spectra of GS-BC and GS-TC did not show peaks corresponding to GS. The absence of peaks in GS-BC and GS-TC indicates complete complexation and/or amorphization of GS takes place. GS completely entrapped in HP-β CD cavity in the presence of PL 188. The similar type of results were found in ternary micro-complex of cefuroxime axetil using HP-β CD and poloxamer 188 [20].

### 3.7. X-ray Diffraction Analysis

**Figure 7:**
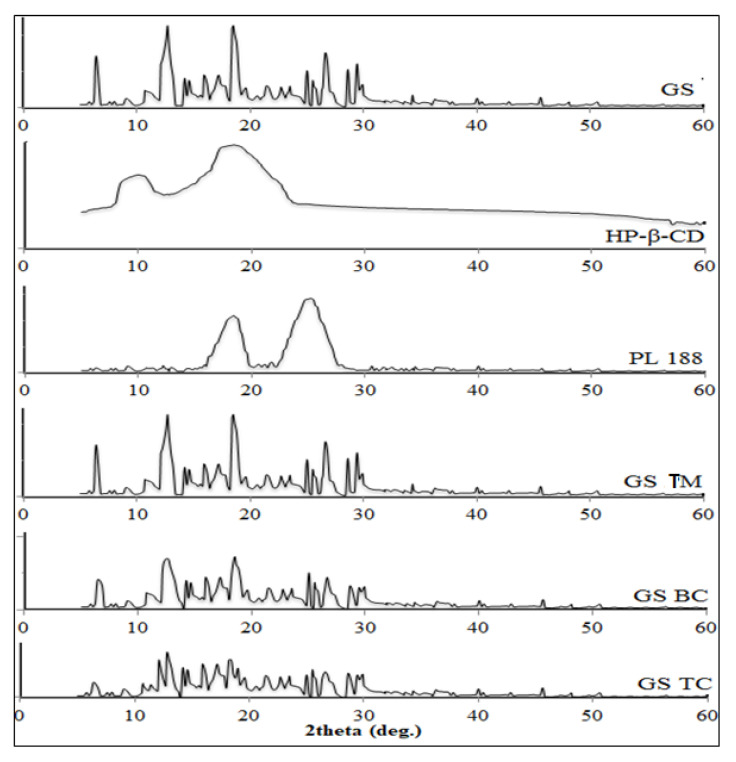
XRD spectra of Genistein, HP-β CD, poloxamer 188, genistein ternary mixture (GS TM), genistein binary complex (GS BC) and genistein ternary complex (GS TC).

Figure 7 shows the XRD spectra of pure GS, HP-β CD, PL 188, GS-PM, GS-BC, and GS-TC. The spectra of pure GS showed the characteristic sharp peak at 6.4°, 12.7°, 18.4°, 25°, 26.6°, 28.6°, and 29.4° assuring its crystalline nature. HP-β CD showed the peaks at 10.1°, 18.5°, and PL 188 depicted at 18.4°, and 25.2°. GS-PM depicted slightly lesser intensity peaks due to the presence of HP-β CD and PL 188 as a carrier. It slightly reduces the crystallinity of GS. The spectra of GS-BC exhibited low-intensity characteristic peaks at 6.4°, 12.2°, 18.4°, 25°. In the case of GS-TC, some peaks of GS disappeared and the available peaks intensity was significantly reduced. The peak height of prepared GS BC and GS-TC was significantly reduced than pure GS. These changes in the property can be interpreted as the complete assimilation of GS in HP-β CD and PL 188.

### 3.8. Nuclear Magnetic Resonance

**Figure 8:**
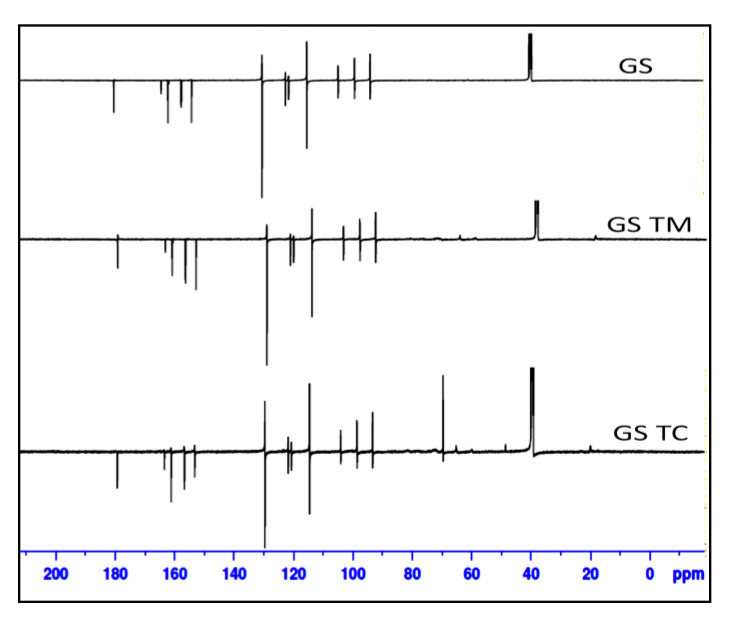
NMR spectra of pure Genistein (GS), genistein ternary mixture (GS-TM) and genistein ternary complex (GS-TC).

^13^C NMR of pure GS, GS-TM and GS-TC were used to check the chemical shift and the data shown in Figure 8 and Table 2. The chemical shift value of pure GS depicted the value of 130.64, 115.54, 99.46,94.22 and 94.15 ppm. These values ascribed for C1, C2, C4, C6 and C8 chromenyl moiety. HP-β CD exhibited a peak of glucose at δ values of 103.14 ppm, whereas PL 188 exhibited peaks for hydrophilic moiety of carbon C attached with the hydroxyl group at δ 64.00 ppm. The chemical shift value of C-O-C- for the hydrophilic moiety was found to be δ 70.60 ppm. On the contrary, the hydrophobic moiety of the carrier PL 188 showed its chemical shift value for C-CO-, having δ value at 72.6 and 77.8 ppm, respectively. The methyl carbon showed its peak at δ 17.1 ppm.

^13^C NMR of GS TM exhibited a slight deviation in peaks for C-4, C-6 and C-8 for chromenyl moiety with a δ value of 99.44, 94.21 and 94.14 ppm, respectively. The δ value for the substituent benzene at C-2′ was found to be at 122.81 ppm. Surprisingly, no peaks were observed for substituent at C-6′ as compared with the values of the pure GS. The glucose peaks of the carrier HP-β CD were also missing from the spectra of GS-TM. The hydrophilic peak for PL 188 was observed with slight change at δ 65.80 ppm which attributes to the carbon C attached with hydroxyl group. A slight deviation was also observed for the peaks of hydrophobic moiety at δ 73.09 ppm which reflects the value of the first carbon for C-CO-. The methyl carbon of the carrier for the physical mixture also exhibited a slight change in its chemical shift value, corresponding to δ 20.20 ppm. Lastly, the GS-PM exhibited all the carbon peaks of the pure drug with the insignificant change in the peaks values for the chromenyl moiety and the substituent carbon of benzene ring. The carrier peak of the HP-β CD glucose was observed in the formulation at δ value of 104.94 ppm. As mentioned earlier, this peak was missing for the spectra of GS-TM. The aforementioned peak exhibited a slight deviation in the peak values when compared with the carrier. With the slight change in the hydrophilic peaks at δ value of 65.80 ppm and hydrophobic peaks at δ value of 70.25 and 72.74 ppm, for PL 188 were also observed in the GS-TC spectra. The peaks of the carriers observed in the spectra may confirm the formation of complexes. 

### 3.9. Scanning Electron Microscope (SEM)

**Figure 9:**
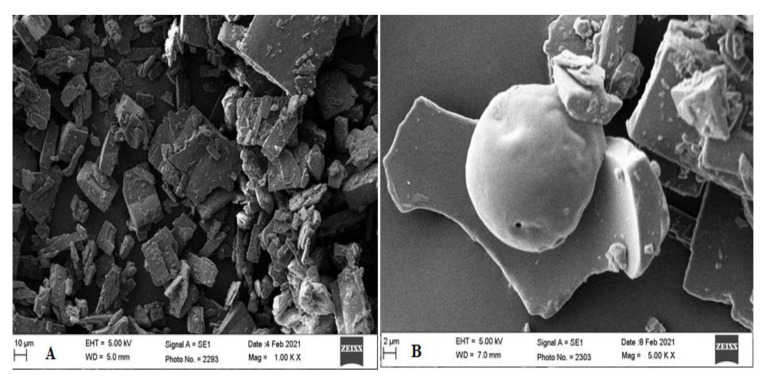
SEM image of (**A**). pure Genistein (GS) and (**B**). Genistein ternary complex (GS TC).

Figure 9 showed the SEM photograph of GS and prepared GS TC to compare the morphological changes. The image of GS exhibited a well-defined crystal structure (Figure 9A). However, in the GS-TC image the morphology of GS has been changed (Figure 9B). The crystal structure entirely disappeared and converted to an amorphous structure with a rough and irregular shape. This is due to the close contact between GS, HP-β CD and PL 188 in the ternary inclusion complex. The particles became entrapped into HP β CD molecules in the presence of PL 188. Therefore, GS may be converted into an amorphous form and may promote dissolution as well as solubility [42,43].

### 3.10. Antioxidant Scavenging Activity

**Figure 10:**
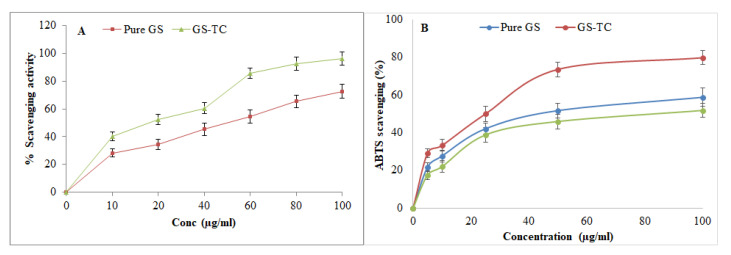
(**A**) DPPH method, (**B**) ABTS scavenging method, Antioxidant results of Genistein (GS) and genistein ternary complex (GS-TC). The data shown as mean ± SD (n = 3).

Figure 10A depicted the comparative antioxidant activity of GS and GS TC using DPPH method. The result showed that the anti-oxidant activity is directly proportional to the concentration of GS. With the increase in GS concentration, the antioxidant activity also increases. A significantly (*p* < 0.05) higher activity was found in the GS TC than pure GS at all concentrations. GS TC exhibited maximum activity at 100 µg/mL concentration (96.23 ± 4.85%), whereas pure GS showed 72.54 ± 5.05% at the same concentration. The significant (*p* < 0.05) high activity of GS that was found in GS TC is due to the enhanced solubility of pure GS in the ternary inclusion complex (HP-β CD and PL 188). The free radical DPPH reacts by different mechanisms with the polyphenols: (a) abstraction of the phenol H atom by DPPH; and (b) electron transfer process from ArOH [44]. GS and DPPH have an aromatic ring in their structure and have poor water solubility. The enhanced antioxidant activity was achieved after enhancing the water solubility with HP-β CD and PL188 ternary complex. From the results, it can be concluded that after complex formation the antioxidant property of GS is enhanced.

The antioxidant activity results revealed the potential antioxidant activity of GS using ABTS method (Figure 10B). The result showed concentration-dependent activity in the concentration between 5–100 µg/mL. There is a significant (*p* < 0.05) difference in the activity that was observed between the pure GS and GS-TC. The maximum effect was observed at the 100 µg/mL concentration. There was a non-significant difference in the activity that was observed at 50 µg/mL (73.67 ± 4.11) and 100 µg/mL (79.88 ± 3.98) in GS-TC. The activity was found to be similar to the DPPH activity. The standard BHT also showed antioxidant potential, but its activity was closer to pure GS. GS-IC showed higher activity due to the enhanced solubility of GS after complexation. The result was found to be similar to DPPH activity.

### 3.11. Cytotoxicity Study

**Figure 11:**
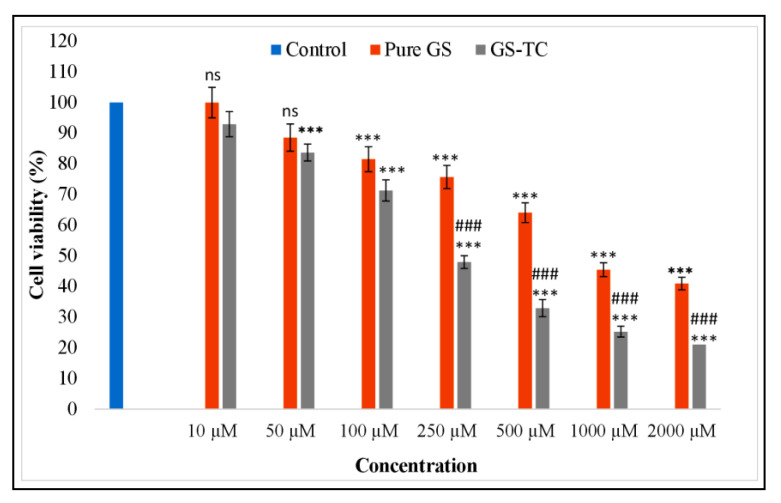
Cytotoxicity study result of Genistein (GS) and prepared genistein ternary complex (GS TC). The data shown as mean ± SD (n = 3). Difference was considered significant if *p* < 0.05. ns = not significant when compared with control; ### *p* < 0.01 when compared between pure GS and GS TC. *** = *p* < 0.001 when compared with control.

The comparative cell viability data of pure GS and GS TC were depicted in Figure 11. The study revealed significant effects on the viability of MCF 7 cells at higher concentrations. The study was performed with MTT at different concentrations of test samples. It was observed that as the GS concentration increases the cell death also increases. A significant difference in the cell viability was observed at all concentrations of pure GS and GS TC. The GS TC showed significant effect at 250 µM (47.93 ± 2.11%), 500 µM (32.91 ± 1.59%), 1000 µM (25.24 ± 2.43%), and 2000 µM (21.02 ± 1.23%) than pure GS at 250 µM (75.68 ± 3.12%), 500 µM (64.04 ± 2.76%), 1000 µM (45.44 ± 1.65%), and 2000 µM (40.95 ± 3.67%). The higher activity of GS TC is due to the greater solubility of GS in the presence of HP β CD and PL 188 (solubilization properties). At lower concentrations, there was non-significant (ns) variation observed in comparison to the control. As the concentration of GS increases, the greater activity was found in GS TC. At 250 µM, 500 µM, 1000 µM, 2000 µM concentration, a highly significant (###, *p* < 0.01 and ***, *p* < 0.001) difference was observed. GS TC exhibited a significant (*p* < 0.001) lesser IC50 value (225 µM) than the pure GS (480 µM). From the results, we can conclude that prepared GS TC significantly reduced the IC50 value as well as high cytotoxicity to the cancer cell.

### 3.12. Antimicrobial Activity

The antimicrobial activity of GS and GS TC was done by the cup-plate method. ZOI of pure GS against S. aureus and B. subtilis was found to be 11.54 ± 0.24 mm and 9.34 ± 0.43 mm, respectively. However, GS TC exhibited ZOI of 17.65 ± 0.26 mm and 14.76 ± 0.31 mm against S. aureus and B subtilis, respectively. HP-β CD—PL 188 mixture also showed slight antibacterial activity. ZOI was found to be 6.45 ± 0.98 mm and 5.55 ± 1.14 mm against S. aureus and B subtilis, respectively. In the case of GS TC, it depicted significantly (*p* < 0.05) better antimicrobial efficacy than pure GS. The high antimicrobial efficacy of GS in GS TC is due to the high solubility of GS in the inclusion complex. The presence of a high concentration of GS from the complex leads to high internalization in bacteria, which inhibited nucleic acid synthesis [45]. The enhanced antibacterial activity of GS may be credited with improving the solubility using the biocompatible, nontoxic HP β CD.

## 4. Conclusions

The inclusion complexes using HP-β CD and PL 188 were prepared by a microwave irradiation method. The prepared complexes were evaluated by FTIR, XRD, DSC, NMR, and SEM, and the results revealed that GS was properly incorporated inside the cavity of HP-β CD. The prepared inclusion complex showed improvement in the solubility and dissolution of GS. The enhancement in dissolution supports the enhanced results of DPPH radical scavenging activity and antimicrobial activity against S. aureus and B. subtilis. The prepared inclusion complex showed a better cytotoxicity effect against the MCF-7 breast cancer cell line due to the marked enhancement in the solubility. Finally, it was concluded that GS TC might be a good approach for the enhancement in solubility problems associated with poorly soluble GS.

## Figures and Tables

**Table 1:** Formulation composition of inclusion complex of GS.

Binary System		Ternary System	
Binary physical mixture (GS BM)	Binary inclusion complex (GS BC)	Ternary physical mixture (GS TM)	Ternary inclusion complex (GS TC)
GS: HP β CD	GS: HP β CD	GS: HP β CD: PL188	GS: HP β CD: PL188
1:1	1:1	1:1:0.5 *	1:1:0.5 *

0.5% * *w*/*w* PL188 added in ternary inclusion complex.

**Table 2:** NMR spectra value of pure genistein (GS), poloxamer 188 (PL 188), HP-β cyclodextrin (HP-β CD), genistein ternary mixture (GS-PM) and genistein ternary complex (GS-TC).

Genistein (GS)	Poloxamer 188 (PL 188)	Hydroxy Propyl Beta Cyclodextrin (HP-β CD)	Genistein Ternary Mixture (GS-TM)	Genistein Ternary Complex (GS-TC)
^13^C NMR Spectral Analysis				
^13^C NMR (176 MHz, DMSO-d_6_) *δ* ppm: 130.64 (C-1), 115.54 (C-2), 99.46 (C-4), 94.22 (C-6) and 94.15 (C-8) of chromenyl moiety, 122.84 (C-2′), 122.78 (C-6′) benzene ring.	^13^C NMR (176 MHz, DMSO-*d_6_*) *δ* ppm: 64 (C of CH_2_-OH),70.6 (C-O-C),72.6 (CH), 17.1 (CH_3_), 77.8 (CH_2_-CH-)	^13^C NMR (176 MHz, DMSO-*d_6_*) *δ* ppm: 103.14(Glucose)	^13^C NMR (176 MHz, DMSO-*d_6_*) *δ* ppm: 122.81(C-2′) of the benzene ring, 99.44 (C-4), 94.21 (C-6) and 94.14 (C-8) of chromenyl moiety, 65.80 (C of CH_2_-OH), 73.09 (CH), 20.20 (CH_3_)	^13^C NMR (176 MHz, DMSO-*d_6_*) *δ* ppm: 130.64 (C-1), 121.68 (C-6′) and 124.19(C-2′) of the benzene ring, 115.53 (C-2), 99.44 (C-4), 94.21 (C-6), 94.14 (C-8) of chromenyl moiety, 65.80 (C of CH_2_-OH), 70.25 (C-O-C), 72.74 (CH), 104.94 (Glucose), 20.20 (CH_3_).

## Data Availability

Not applicable.
